# Fungicide Sensitivity Shifting of *Zymoseptoria tritici* in the Finnish-Baltic Region and a Novel Insertion in the *MFS1* Promoter

**DOI:** 10.3389/fpls.2020.00385

**Published:** 2020-04-15

**Authors:** Andres Mäe, Sabine Fillinger, Pille Sooväli, Thies Marten Heick

**Affiliations:** ^1^Department of Plant Protection, Estonian Crop Research Institute, Jõgeva, Estonia; ^2^Université Paris-Saclay, INRAE, AgroParisTech, UMR BIOGER, Thiverval-Grignon, France; ^3^Department of Agroecology, Aarhus University, Slagelse, Denmark

**Keywords:** azoles, MDR, SDHI, septoria tritici blotch, transposon, QoI

## Abstract

Septoria tritici blotch (STB) is caused by the ascomycete *Zymoseptoria tritici* and one of the predominating diseases in wheat (*Triticum aestivum*) in Europe. The control of STB is highly reliant on frequent fungicide applications. The primary objective of this study was to assess sensitivity levels of *Z. tritici* to different fungicide groups. The fungicides included in this study were epoxiconazole, prothioconazole-desthio, tebuconazole, and fluxapyroxad. A panel of 63 isolates from Estonia, Latvia, and Lithuania, and 10 isolates from Finland were tested. Fungicide sensitivity testing was carried out as a bioassay analyzing single pycnidium isolates on different fungicide concentrations. The average EC_50_ value in Baltic countries and Finland to epoxiconazole was high ranging from 1.04 to 2.19 ppm. For prothioconazole-desthio and tebuconazole, EC_50_ varied from 0.01 to 0.24 ppm, and 1.25 to 18.23 ppm, respectively. The average EC_50_ value for fluxapyroxad varied from 0.07 to 0.33 ppm. To explain the range of sensitivity, the samples were analyzed for *CYP51* and *Sdh* mutations, as well as *cytb* G143A, *CYP51* overexpression, and multidrug resistance (MDR). Frequencies of *ZtCYP51* mutations D134G, V136A/C, A379G, I381V, and S524T in the Finnish-Baltic region were lower than in other European countries, but have increased compared to previous years. The frequency of *cytb* G143A conferring strobilurin resistance also augmented to 50–70% in the *Z. tritici* populations from Estonia, Finland, Latvia, and Lithuania. No *Sdh* mutations were found in this study, and neither strains of MDR phenotypes. However, we found a strain harboring a previously unknown transposon insertion in the promoter of the *MFS1* gene, involved in drug efflux and multi-drug resistance. This new insert, however, does not confer an MDR phenotype to the strain.

## Introduction

*Zymoseptoria tritici* is the causal agent of septoria tritici blotch (STB), the most devastating leaf disease on wheat (*Triticum aestivum*) worldwide (O’Driscoll et al., 2014; Fones and Gurr, 2015). The disease thrives best under humid-temperate conditions and can have several disease cycles per season. STB is present throughout Europe but is the prevalent disease in regions that offer conducive climatic conditions such as Ireland, the United Kingdom, or Northern Germany (Jørgensen et al., 2014). The disease incidence of STB decreases toward South and North-East Europe (Garnault et al., 2019), where STB competes with other leaf blotch diseases, for instance, *Parastagonospora nodorum* and *Pyrenophora tritici-repentis* (Heick et al., 2017b). Agricultural practices have a direct impact on disease severity. Whereas early sowing of winter wheat and minimum tillage favor epidemics of STB, the use of varietal resistance and a delay of sowing help mitigate the primary inoculum at the beginning of the season, and thus disease severity in the following year. Despite recent achievements in breeding and a focus on non-chemical practices (Gladders et al., 2001; Brown et al., 2015), STB control is highly reliant on frequent and timely applications of fungicides. Yield losses can amount to up to 30–50% if the disease is not efficiently controlled (Jørgensen et al., 2014). Currently, three main fungicide groups are available for STB disease control: (1) quinone outside inhibitors (QoI), (2) 14α-demethylase inhibitors (DMI), and (3) succinate dehydrogenase inhibitors (SDHI). Compounds of those three groups have been used for many years now and have efficiently reduced the impact of STB. Nevertheless, field efficacies of many active ingredients belonging to those groups have reduced due to fungicide resistance in recent years (Blake et al., 2018; Kildea et al., 2019).

Population genetic studies based on mark–release–recapture experiments conducted in the field indicated that pathogen populations might change significantly over a single growing season in response to host genotypes (Zhan et al., 2002). The development of fungicide resistance has been attributed to several molecular mechanisms, including alterations and overexpression of the target-site and the pathogen’s ability to lower the amount of fungicide in the cell through overexpression of efflux pumps (Cools and Fraaije, 2013). Resistance to all single-site fungicides is present in most *Z. tritici* populations, to which degree, however, may greatly vary locally. Mutations in the target gene of a fungicide are the most common mechanisms of fungicide resistance in *Z. tritici* (Mullins et al., 2011). Fungicide resistance can occur in a disruptive manner or stepwise.

Resistance to QoI fungicides arose promptly after the introduction of this mode of action (MoA) in the early 2000s, associated with point mutations in the mitochondrial *cytochrome b* gene (*cytb*). Isolates carrying F129L or G137R express moderate (partial) resistance and are comparably somewhat sporadic in *Z. tritici* populations. In contrast, point mutation G143A confers full resistance and dominates in current *Z. tritici* populations. Consequently, QoI fungicides are no longer effective against *Z. tritici* in most European countries (Fraaije et al., 2005; Sierotzki et al., 2006).

Since their introduction in the 1970s, azoles have become essential components of plant disease control in the fields because of their wide-ranging efficacy against many agriculturally important diseases (Russell, 2005). Prolonged and intensive usage of agricultural azole fungicides in crop protection has been the main driver in the emergence of azole resistance in fungi. Several molecular mechanisms play a role in reduced azole sensitivity. The most common mechanism is alterations in the *CYP51* gene leading to amino acid changes of the CYP51 enzyme. To date, over 30 different amino acid alterations (substitutions and deletions) have been identified in the CYP51 protein of modern *Z. tritici* populations, and over 30 different genotypes have been registered so far (Cools and Fraaije, 2013; Huf et al., 2018). Mutations leading to exchanges D134G, V136A/C/G, A379G, I381V, S524T, and deletions or mutations at nucleotides coding for amino acids 459–461 are currently claimed to have the highest effect on the sensitivity to azoles (Cools et al., 2011; Wieczorek et al., 2015). The emergence of S524T in field populations of *Z. tritici* has caused particular concern as prothioconazole, along with epoxiconazole, is one of the two remaining azole fungicides still highly effective against STB (Cools et al., 2011). Mutations can occur in combination, and the presence or absence of a single mutation can render an isolates resistant. Furthermore, Cools et al. (2012) described an insertion of 120 bp in the *CYP51* gene that leads to overexpression of the gene and, consequently, an increased azole tolerance.

After the rapid development of resistance against QoI and DMI fungicides, a newer generation of SDHI for cereal diseases was launched in the last few years that are very effective in controlling STB (Sierotzki and Scalliet, 2013). Several target mutations have already been described both in the laboratory and in the field, which can lead to SDHI resistance. For example, C-T79N can be found alone or in combination with C-I29V or with both C-N33T, C-N34T, and C-H152R (Dooley et al., 2016). Several point mutations in the *Sdh* subunits have been associated with high EC_50_ values: B-N225I/T, B-H267X, B-T268I, B-I269V, C-N86S, C-N86K, C-T79N, C-T79I, C-W80S, C-G90R, and C-H152R (Rehfus et al., 2018). Unlike the *CYP51* mutations, haplotypes with more than one *Sdh* mutation have rarely occurred in nature yet.

In addition to the target-site-specific mutations, Leroux and Walker (2011) described a third mechanism, rendering *Z. tritici* phenotypes resistant to several MoA, a phenotype considered as multi-drug-resistant (MDR). Those strains were later associated with an enhanced active fungicide efflux (Omrane et al., 2015). Three different types of inserts in the promoter region of the “major facilitator gene” (*MFS1*) were identified in MDR field strains leading to overexpression of this gene. In the current *Z. tritici* population, the number of MDR strains are increasing and are frequently found in Western Europe (Kildea et al., 2019). In Northern Europe, strains harboring one of the inserts are present, but in low frequency (Heick et al., 2017b).

In Baltic countries, a wider range of active ingredients in fungicides is available than in other Northern European countries. Over a long period now, the Baltic *Z. tritici* populations have been exposed to different MoA; however, a systematic resistance testing of *Z. tritici* has not been carried out. The overall objective of this study was to describe the current fungicide resistance situation of *Z. tritici* in the Baltic region.

## Materials and Methods

### Isolate Collection

A total of 73 single pycnidium isolates of *Z. tritici* were produced, 29 of which from Estonia, 4 from Latvia, 30 from Lithuania, and 10 from Finland in 2018. Leaf samples were collected from the upper two leaves layers, mainly from untreated plots. The leaves were placed in a petri dish on moist filter paper without prior surface sterilization. After 24 h incubation at room temperature, cirrhi from single pycnidia were transferred onto potato dextrose agar, amended with 0.01% streptomycin, using a sterile needle. Single spore colonies appeared after 6 days of incubation at 20°C and 12 h white light/12 darkness. Spores were conserved in 20% glycerol at −80°C.

### Determination of EC_50_

Spore suspensions were produced by scraping off 6-day-old *Z. tritici* spores and transferring them into sterile, demineralized water. The suspensions were vortex-mixed in 10 ml Falcon tubes for 10 min for homogenization. Spore concentrations were adjusted to 2.5 × 10^4^ spores ml^–1^. Epoxiconazole, tebuconazole, and prothioconazole-desthio (all Sigma-Aldrich, St. Louis, MO, United States) were mixed separately with 2 × potato dextrose broth to obtain the following final microtiter plate fungicide concentrations (mg l^–1^): 10, 3.3, 1.0, 0.3, 0.1, 0.03, 0.01, and 0 for epoxiconazole and 90, 30, 10, 3.3, 1.0, 0.3, 0.1, and 0 for prothioconazole-desthio and tebuconazole. One hundred microliter of spore suspension and 100 μl fungicide solution were added to a nunc^TM^ 96-deep well microtiter plate (Thermo Fisher Scientific, Roskilde, Denmark). Technical duplicates of each isolate were performed on the same plate, and Dutch isolate IPO323 (azole-sensitive) and the Irish isolate OP15.1 (moderately azole-resistant) were included as references. Microtiter plates were wrapped in aluminum foil and incubated in the dark at 20°C for 6 days. The plates were visually checked for bacterial and fungal contamination before the analysis in an iMark^TM^ Microplate Absorbance Reader (Bio-Rad, Copenhagen, Denmark) at wavelength 620 nm. The fungicide dose reducing growth in the microplate wells by 50% (EC_50_) was determined by non-linear regression (curve-fit) using GraphPad Prism (GraphPad Software, La Jolla, CA, United States). Resistance factors were calculated based on the reference EC_50_ values for IPO323. The EC_50_ values for fluxapyroxad (Sigma-Aldrich, St. Louis, MO, United States) were determined correspondingly using a 10-fold dilution series starting at 3 ppm.

### Sequencing of Target Site Mutations

All isolates were tested for target mutations for azole, SDHI, and strobilurin fungicides. The presence of *CYP51* mutations L50S, D134G, V136A/C, G379A, I381V, and S524T and *cytb* mutation G143A conferring strobilurin resistance were determined using Kompetitive Allele Specific PCR (KASP) (LGC Genomics, Teddington, United Kingdom) genotyping previously described by Kildea et al. (2014). All reactions were carried out in an Applied Biosystems Viia^TM^ 7 Real-time PCR system machine (Thermo Fisher Scientific, Denmark) according to the manufacturer’s protocol.

Sequences of the *Sdh* sub-units B, C, D were obtained using the protocol by Rehfus et al. (2018). PCR reactions were performed utilizing the GoTaq Flexi DNA Polymerase kit (Promega, Madison, WI, United States) in a 25 μl volume containing 10.9 μl Gibco water, 5.0 μl 5× GoTaqFlexi PCR buffer, 1.5 mM MgCl_2_, 125 μM of each dNTP, and forward primer and reverse primer (both 10 μM), 1 unit GoTaqFlexi DNA polymerase (Promega, Madison, WI, United States), and 1.0 μl DNA (approximately 5 ng μl^–1^). The amplification was performed using the following conditions: 95°C for 5 min, followed by 35 cycles of 95°C for 30 s, 62°C for 30 s, 72°C for 30 s, with a final extension of 7 min at 72°C. The PCR products were purified and sequenced by Macrogen Europe BV (Amsterdam, Netherlands). Sequence alignment was performed using CLC WorkBench 7 (QIAGEN, Aarhus, Denmark).

### Testing for Potential Overexpression of the *CYP51* and *MFS1* Genes

All isolates were investigated for the presence of inserts in the *CYP51* promoter region, conferring CYP51 overexpression (Cools et al., 2012). PCR reactions were performed using the GoTaq Flexi DNA Polymerase kit (Promega, Madison, WI, United States) in a 25 μl volume containing 10.9 μl Gibco water, 5.0 μl 5× GoTaqFlexi PCR buffer, 1.5 mM MgCl_2_, 125 μM of each dNTP, and forward primer Mg51-proF and reverse primer Mg51-seqR (both 10 μM), 1 unit GoTaqFlexi DNA polymerase, and 1.0 μl DNA (approximately 5 ng μl^–1^). The PCR conditions were 95°C for 5 min, followed by 35 cycles of 95°C for 30 s, 62°C for 30 s, 72°C for 30 s, with a final extension of 7 min at 72°C.

To screen for inserts in the promoter region of the *MFS1* gene, PCR reactions were performed using primer pair MFS_2F/MFS_4R designed by Omrane et al. (2015). PCR reactions were set up in a 25 μl volume containing 19.75 μl Gibco water, 2.0 μl buffer, 1.25 μl dNTP mix forward primer and reverse primer (both 10 μM), and 1.0 μl DNA. The reactions were run according to the following protocol: 35 cycles of 95°C for 30 s, 62°C for 30 s, 72°C for 30 s, with a final extension of 7 min at 72°C.

All PCR reactions in this study were performed in an Applied Biosystems 2720 Thermal Cycler. The samples were loaded on a 1.5% agarose gel containing SYBR^®^ stain (Thermo Fisher Scientific, Denmark) and run for 45 min at 100 V.

### *MFS1* Insert Sequence Analysis

The PCR fragment of strain 18-Zt-EE-06-03 obtained with the primer couple MFS_2F/MFS_4R was Sanger sequenced (Eurofins Genomics) with the same primers and the additional sequencing primer Estland-sample_LF (5′ TGGTGTTTCCATGCGTTTAG 3′). Output sequences were trimmed and assembled using the CodonCode Aligner software v9.0.1 (CodonCode Corp., Centerville, MA, United States). The insert sequence and surrounding *MFS1* promoter sequence have been deposited at Genbank under accession number MN813065. The insert sequence was used for database searches using blastn, blastx (Altschul et al., 1990) at NCBI^1^ against non-redundant databases. The peptide translation of the longest ORF was analyzed using PSI-blast and DELTA-blast at NCBI. Inverted repeats were identified using the einverted algorithm^2^. Searches for annotated transposable elements (TE) were performed as blastn-searches against the TE database Repbase (Kapitonov and Jurka, 2008).

## Results

### Azole Sensitivity Testing and CYP51 Sequence Analysis of Baltic *Z. tritici* Strains

Seventy-three *Z. triti*ci isolates were sampled in Estonia (29), Latvia (4), Lithuania (30), and Finland (10) during 2018. The individual pycnidium isolates were investigated for sensitivity to epoxiconazole, prothioconazole-desthio, and tebuconazole in order to calculate EC_50_ value for each isolate. In 2018, the average EC_50_ value for epoxiconazole was 1.73 ppm, with single isolates ranging from 0.35 to 8.29 ppm. The average EC_50_ was highest in Estonia (2.19 ppm) but lower in Latvia (1.97 ppm), Lithuania (1.67 ppm), and Finland (1.04 ppm). The average resistance factor (RF; mean EC_50_ of the resistant population)/(mean EC_50_ of the reference isolate IPO323) for epoxiconazole was 69 (reference isolate IPO323: 0.02–0.03 ppm) ranging from 42 in Finland to 87 in Estonia, 67 in Lithuania, and 79 in Latvia (Table 1). The average EC_50_ value for prothioconazole-desthio was 0.14 ppm, with single isolates ranging from 0.01 to 2.0 ppm. The average EC_50_ was highest in Lithuania (0.24 ppm) but lowest Estonia (0.14 ppm), Latvia (0.13 ppm), and Finland (0.06 ppm). The average RF for prothioconazole-desthio was 14 (reference isolate IPO323: 0.01 ppm) ranging from 6 in Finland to 24 in Lithuania, 13 in Latvia, and 11 in Estonia. The average EC50 value for tebuconazole was 8.45 ppm, with single isolates ranging from 0.13 to 59 ppm. The average EC_50_ was highest in Estonia 18.23 ppm (0.13–59 ppm) but lower Lithuania 9.18 ppm (0.8–48 ppm), Finland 5.13 ppm (0.3–18.2 ppm) and Latvia 1.25 ppm (0.34–2.4 ppm). The average RF for tebuconazole was 211 (reference isolate IPO323: 0.041 ppm) ranging from 31 in Latvia to 456 in Estonia, 128 in Finland, and 230 in Lithuania (Table 1).

**TABLE 1:** Summary of average EC_50_ (ppm) values and resistance factors (RF)^a^ for epoxiconazole, prothioconazole-desthio, and tebuconazole assessed for *Z. tritici* in Estonia, Finland, Latvia, and Lithuania.

**Country**	**Epoxiconazole**		**Prothioconazole-desthio**		**Tebuconazole**	
	**Average**	**RF**	**Average**	**RF**	**Average**	**RF**
Estonia (*n* = 29)	2.19	87	0.14	11	18.23	456
Finland (*n* = 10)	1.04	42	0.06	6	5.13	128
Lithuania (*n* = 30)	1.67	67	0.24	24	9.18	230
Latvia (*n* = 4)	1.97	79	0.13	13	1.25	31
Average	1.73	69	0.14	14	8.45	211
Reference IPO323	0.02–0.03		0.01		0.04	

*^*a*^Resistance factor: (mean EC_50_ of resistant population)/(mean EC_50_ of reference isolate IPO323).*

**FIGURE 1:**
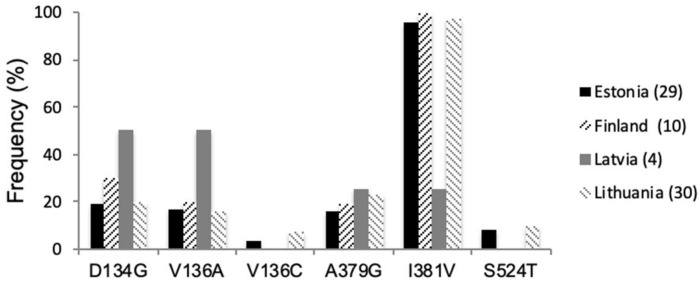
*CYP51* mutation frequencies (%) in *Z. tritici* populations from Estonia, Finland, Latvia, and Lithuania in 2018.

Several point mutations in the *CYP51* gene have been associated with elevated EC_50_ values for azoles. Sequencing analysis of all selected isolates showed the presence of *CYP51* point mutations D134G, V136A/C, I381V, and S524T in *Z. tritici*, associated with increased fungicide resistance to the currently widely used azoles (Figure 1). Mutation I381V continued to dominate throughout the region and is present in frequencies of 96–100%, except for Latvia, where this mutation was present at a lower rate, 25%, respectively. Grouping of *Z. tritici* isolates from different Baltic countries revealed 14 different genotypes (Table 2). The substitution S524T, found in Estonian and Lithuanian samples, was always combined with V136A and I381V and in some isolates, these three substitutions were combined with L50S and D134G (Table 2). The frequencies for mutations L50S, D134G, V136A/C, and A379G, all of which have recently emerged in the European *Z. tritici* population, varied considerably. The only single substitutions of CYP51 found in the in Baltic-Finnish isolates were V136A and I381V.

**TABLE 2:** Amino acid polymorphisms in the *CYP51* gene from *Z. tritici* field isolates collected in the Finnish-Baltic region in 2018.

**Origin**	**Position of amino acid polymorphism**					
	**L50**	**D134**	**V136**	**A379**	**I381**	**T524**
EE, LT	S	G	V	A	V	T
EE	S	D	A	A	V	T
LT	S	G	A	A	V	T
EE, LT	L	D	A	A	V	T
EE, FI	S	G	A	A	V	S
LT	L	G	A	A	V	S
EE, LT	S	D	V	G	V	S
LT. FI	L	G	A	A	V	S
EE, FI, LT, LV	L	D	V	G	V	S
FI	L	G	V	A	V	S
EE, FI	S	D	V	A	V	S
LV	L	G	A	A	V	S
EE, FI, LT	L	D	V	A	V	S
LV	L	D	A	A	I	S
						

### Identification of G143A Mutations *Z. tritici* Strains Isolated in Finnish-Baltic Region

So far, G143A has been found in all isolates with high resistance levels to strobilurins (Fraaije et al., 2005; Sierotzki et al., 2006). All the isolates collected during 2018 were subsequently screened using the qPCR assay. The allele A143 was found in 41 of the 73 isolates (56%; Table 3). Analysis of the results showed that the frequencies of this mutation were at a high level throughout the Finnish-Baltic region: Estonia, Finland, Latvia (50%), and Lithuania (73%) (Table 3).

**TABLE 3:** Frequency of G143A mutations in *Z. tritici* collected in 2018 across the Baltic-Finnish region.

**Country**	**Frequency of G143A mutation**		
	**G143A**	**G143**	**% G143A**
Estonia	14	14	50
Finland	5	5	50
Latvia	2	2	50
Lithuania	22	8	73

### Sensitivity of Baltic-Finnish Isolates to Fluxapyroxad

The sensitivity profiles of all 73 single-spore isolates from the Finnish-Baltic region to SDHI fluxapyroxad were measured. The average EC_50_ value for fluxapyroxad was 0.2 ppm, with single isolates ranging from 0.07 to 0.33 ppm. The average EC_50_ was highest in Lithuania 0.33 ppm (0.01–1.00 ppm) but lower in Latvia 0.23 (0.05–0.49 ppm), Estonia 0.18 ppm (0.04–1.12 ppm), and Finland 0.07 ppm (0.03–0.27 ppm; Table 4). The average RF for fluxapyroxad was 7 (reference isolate IPO323: 0.03 ppm) ranging from 11 in Lithuania to 8 in Latvia, 6 in Estonia, and 2 in Finland (Table 4). Several point mutations in the *Sdh* subunits B, C, and D have been associated with elevated EC_50_ values. In this study, we did not detect any alterations in the *Sdh* subunits.

**TABLE 4:** Summary of measured EC_50_ (ppm) values and resistance factors (RF) for fluxapyroxad assessed for *Z. tritici* in Estonia, Finland, Latvia, and Lithuania.

**Country**	**Fluxapyroxad**	
	**Average**	**RF**
Estonia (*n* = 29)	0.18	6
Finland (*n* = 10)	0.07	2
Lithuania (*n* = 30)	0.23	8
Latvia (*n* = 4)	0.33	11
Average	0.20	7
Reference IPO323	0.03	

### Insertion in the *CYP51* Promoter Region

**FIGURE 2:**
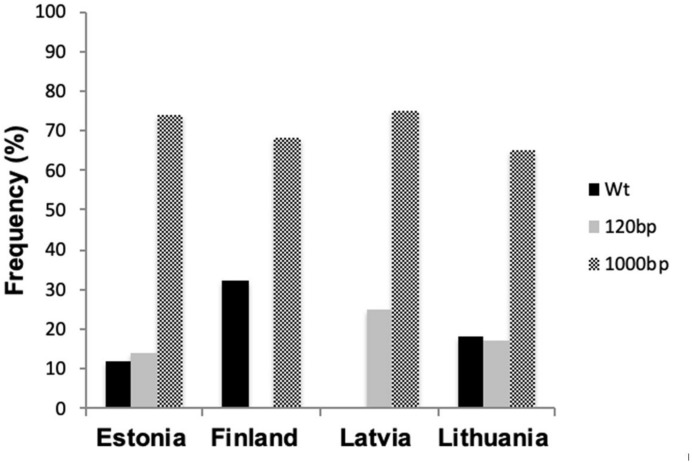
Promoter inserts frequency (%) of the *CYP51* gene of Estonian, Finnish, Latvian, and Lithuanian *Z. tritici* strains.

From the Finnish-Baltic region, in 18% of all isolates tested a PCR fragment of 334 bp was detected, indicating that these isolates had no insert (wild-type). From four Estonian isolates (14%), one Latvian isolate (25%), and five Lithuanian isolates (17%), a PCR fragment of approximately 450 bp was amplified, indicating that these isolates had 120 bp insert in the promoter region as described by Cools et al. (2012). In Estonian, Finnish, Latvian, and Lithuanian isolates, the 866 bp insert, previously described by Chassot et al. (2008),Omrane et al. (2015), and Kildea et al. (2019), was present in 65–75% (Figure 2).

### Detection of Inserts in the *MFS1* Promoter as Potential Drivers of Multi-Drug Resistance

We then searched for potential MDR isolates as described by Omrane et al. (2015). We, therefore, amplified in all *Z. tritici* isolates a promoter fragment of the *MFS1* gene encoding a membrane transporter of the major facilitator superfamily (Roohparvar et al., 2007, 2008) involved in MDR in *Z. tritici* field strains (Omrane et al., 2015, 2017), using the primer couple published by Omrane et al. (2015). All but one isolate gave rise to the “sensitive” allele of 500 bp. Only isolate 18-ZT-EE-06-03 led to an amplicon of >2 kb. Sensitivity assays to the squalene epoxidase inhibitors tolnaftate and terbinafine, used as monitoring molecules to detect MDR (Leroux and Walker, 2011), revealed that the 18-Zt-EE-06-03 isolate was not resistant to these molecules and therefore potentially not MDR (data not shown).

**FIGURE 3:**
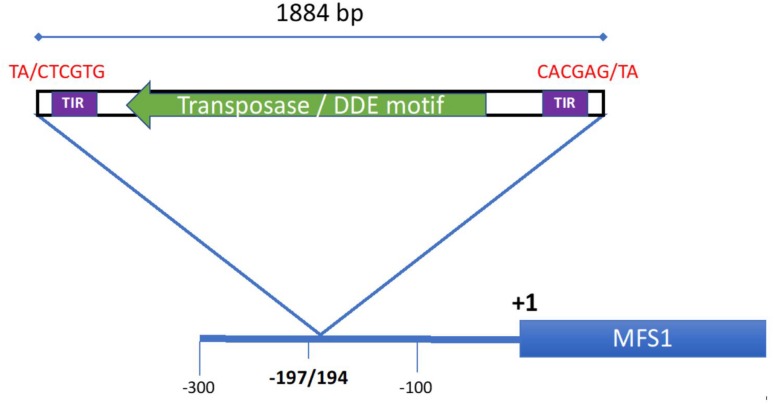
Promotor structure of the *MFS1 locus* in strain 18-ZT-EE-06-03. The promotor insert with its location is indicated by the blue triangle. The inverted target site duplications at the insertion site are indicated in red in 5′–3′ direction. A terminal inverted repeat (TIR) sequence of 62 bp as detected by the “inverted” algorithm is indicated by the violet boxes. An ORF of 1032 bp with strong similarity to transposases is indicated by the green arrow.

Sequence analysis of the promoter amplicon revealed an 1884 bp previously unknown insert at position-194 relative to the Start codon (Figure 3). Blastn searches at Genbank revealed that the sequence was highly similar to sequences found in other *Z. tritici* strains (three copies in strains 3D7 and 1A5, one copy in 3D1, and IPO323, respectively), although at other locations. Blastx searches at Genbank revealed a conserved protein domain, known as DDE superfamily endonuclease involved in DNA transposition. We identified an open-reading-frame (ORF) of 1032 bp (343 amino acids) transcribed from the complementary strand with substantial sequence similarity (>40% identity according to the algorithm used) to transposases as revealed by DELTA-blast (Boratyn et al., 2012) and PSI-blast (Altschul et al., 1997) searches at NCBI. We then searched the Repbase database of eukaryotic TEs for similarity to any known TE, without success. Manual and *in silico* analysis of the promoter insert sequence, following the guidelines proposed by Wicker et al. (2007) for TE analysis, allowed us to detect the target site duplication (TSD) 5′ TA/CTCGTG 3′, inversed at the opposite end, and a rather long terminal inverted repeat of 62 bp (with four mismatches). In recent work, Badet et al. (2020) have annotated TEs in the *Z. tritici* pangenome. Sequences highly similar to the MFS1 promoter insert of strain 18-Zt-EE-06-03 were annotated as DTT_Birute transposon family of the *Tc1-Mariner* superfamily, class II DNA transposons (Oggenfuss and Croll, personal communication) according to the nomenclature proposed by Wicker et al. (2007). The sequence was uploaded to GenBank under accession number MN813065.

## Discussion

The recent decline in field efficacy against STB of azole and SDHI fungicides in Northwestern Europe (Heick et al., 2017b; Kildea et al., 2019) led us to investigate the present resistance situation of *Z. tritici* in the Finnish-Baltic region. Up to now, azoles and SDHI have provided sufficient control in Estonia, Latvia, and Lithuania. In our investigation, we tested single pycnidium isolates of *Z. tritici in vitro* for their sensitivity toward commonly used azoles for STB control. The results confirmed different levels of sensitivity for all isolates tested for the different azoles. The EC_50_ values for epoxiconazole were highest in Estonia and prothioconazole-desthio in Lithuania (Table 1). The average EC_50_ values for epoxiconazole were >1 ppm throughout the region, indicating a general adaption of the Baltic-Finnish *Z. tritici* population to this active ingredient. Compared to the resistance status in 2014, described by Heick et al. (2017b), an apparent sensitivity shift has occurred over the last four years (Table 5). The resistance factors for prothioconazole-desthio were lower than for epoxiconazole and indicated a moderate adaption.

**TABLE 5:** Comparison of average EC_50_ values (ppm) and resistance factors (RF) of epoxiconazole assessed for *Z. tritici* in Estonia, Finland, Latvia, and Lithuania in 2014 and 2018.

**Country**	**Epoxiconazole**			
	**Average**		**RF**	
	**2014**	**2018**	**2014**	**2018**
Estonia	0.07	2.19	7	87
Finland	0.23	1.04	4	42
Lithuania	0.14	1.67	15	67
Latvia	0.16	1.97	17	79
Average	0.15	1.73	16	69

The resistance level of tebuconazole was significantly higher for all isolates tested (Table 1). Resistance to tebuconazole has been widely spread in Europe and attributed to *CYP51* mutation I381V (Fraaije et al., 2007). The field performance of tebuconazole has thus been considerably low in many European regions. Jørgensen et al. (2018a) described, however, a regional variation for some countries where tebuconazole still performed well. Also, in the Baltic countries, tebuconazole is still widely used and provides reasonable STB control in the field. The results from this study, though, suggested an adaption to a more tebuconazole-resistant *Z. tritici* population. Compared to other countries, this adaption has reached the Finnish-Baltic region with a delay.

The first cases of fungicide-adapted strains are usually found in “high-risk” areas with conducive climatic conditions for a fungus. High disease incidence and high inputs of fungicide to control the pathogen lead to a rapid selection for resistant strains (Jørgensen et al., 2018b). Examples for azole resistance development of *Z. tritici* at different paces have been observed in Northwestern Europe and France (Heick et al., 2017b; Garnault et al., 2019). In the United States and Australia, where fungicide inputs are traditionally lower than in Europe, the adaption of *Z. tritici* to azoles is first now occurring (Sykes et al., 2018; McDonald et al., 2019). In Europe, Jørgensen et al. (2018a) showed that a gradient of fungicide resistance from west to east exists. Resistance issues of epoxiconazole and prothioconazole were reported in the United Kingdom from 2008. In Denmark, resistance to epoxiconazole and prothioconazole, both measured as the decline in field efficacy and *in vitro*, had not occurred before 2015. From 2015, field efficacy for both compounds declined noticeably, coinciding with a drastic increase in the frequency of azole-resistant isolates (Heick et al., 2017b). The recent appearance of increasingly more azole-resistant isolates in the Baltic area suggests a similar trend, as seen in Denmark and Sweden. Intensified fungicide monitoring activities for *Z. tritici* in the region will be carried in the coming year to follow up on this development. The development appears to be consistent in all three Baltic countries and Finland. In a study, Vagndorf et al. (2018) compared the *Z. tritici* isolates from the different Scandinavian and Baltic countries in 2014 using AFLP markers, and found two distinct populations: a Danish-Swedish and a Baltic-Finnish. The existence of a single Baltic-Finnish *Z. tritici* population might explain a delayed and simultaneous change in fungicide sensitivity.

Several studies have previously demonstrated that applications of fungicide select for mutations both *in vitro* and in the field (Leroux et al., 2007; Wieczorek et al., 2015; Heick et al., 2017a). Several studies identified single nucleotide polymorphisms in the *CYP51* gene being the primary force behind azole resistance (Cools et al., 2011; Leroux and Walker, 2011; Cools and Fraaije, 2013). A specific combination of the *CYP51* mutations determines the final sensitivity of an individual *Z. tritici* strain. However, as not all alterations are equally important, single frequencies of specific *CYP51* mutations give a proper indication for the selection status of a population.

In this study, we investigated single pycnidium *Z. tritici* isolates sampled in the Finnish-Baltic region in 2018 for the occurrence of most important *CYP51* mutations D134G, V136A/C, A379G, I381V, and S524T (Leroux et al., 2007; Cools and Fraaije, 2013) to determine the resistance stage of this population. The leaf samples derived primarily from untreated plots to determine fungicide resistance in the absence of fungicides. I381V remains the most predominant mutation in all Baltic countries (Heick et al., 2017b; Vagndorf et al., 2018); only in Latvia, this mutation was present at low levels in 2018 (Figure 1 and Table 6). Point mutation A379G, known to occur exclusively in combination with I381V, was present in all countries and showed an minor increase compared to 2014 (Heick et al., 2017b). D134G and V136A, which were absent or present at a low level in the Finnish-Baltic region in 2014, have increased over five years up to a level of 50% in Lithuania (Figure 1 and Table 6). V136C and S524T, which, especially in combination, have a significant impact on azole sensitivity (Cools et al., 2011), were present in Estonia and Lithuania at low levels of less than 10% (Figure 1). All the isolates tested from the Finnish-Baltic region had at least one mutation in the *CYP51* gene. The *Z. tritici* wild-type, which was still present in 2014, seems thus to be replaced (Heick et al., 2017b). The evolution of *CYP51* mutations in the Baltic-Finnish *Z. tritici* population starts more to resemble that in Denmark and Sweden (Heick et al., 2017b; Vagndorf et al., 2018). This development is an indication that the evolution of the *CYP51* gene has reached the Northeastern parts of Europe. This assumption is supported by the fact that only 2 of 14 haplotypes found in the Finnish-Baltic region showed a single mutation in the *CYP51* gene (Table 2). In addition to target site mutation, the overexpression of the target gene can confer tolerance toward fungicides. In the Baltic *Z. tritici* population, we detected the 120 bp insertion in the promoter region of the *CYP51* gene for the first time (Figure 2). It was first described by Cools et al. (2012) and associated with an overexpression of the *CYP51* gene, increasing a strain’s tolerance to azole fungicides. This insertion has been detected in several *CYP51* haplotypes (Huf et al., 2018), and has increased in frequency in the European *Z. tritici* population in recent years. Presumably, due to a fitness advantage in the presence of fungicides. Furthermore, the 866 bp insertion in the *CYP51* promoter region was found at high frequencies (>70%) in all countries (Chassot et al., 2008). This high number is consistent with studies of other European *Z. tritici* populations (Heick et al., 2017b; Kildea et al., 2019). Kildea et al. (2019) found an overexpression of the *CYP51* gene in the presence of epoxiconazole in a specific CYP51 background. However, the impact of this insert on the *CYP51* gene needs to be further investigated to be entirely understood.

**TABLE 6:** *CYP51* mutations frequencies (%) in *Zymoseptoria tritici* samples from the Finnish-Baltic region in 2014 and 2018.

**Country**	**Year**	**Mutations (%)**					
		**D134G**	**V136A**	**V136C**	**A379G**	**I381V**	**S524T**
Estonia	2014	0	0	0	0	100	0
	2018	19	8	3	16	98	8
Finland	2014	17	8	8	10	77	5
	2018	30	20	0	28	100	0
Lithuania	2014	10	12	5	12	80	5
	2018	20	20	0	23	98	10
Latvia	2014	0	5	0	10	95	0
	2018	50	50	6	25	25	0

The primary mechanism of decreased sensitivity against strobilurin class fungicides is the alteration of *cytb* at location G143 (mutation G143A). Resistance toward QoI fungicide occurred at least four times simultaneously in Europe and spread rapidly throughout Europe (Fraaije et al., 2005; Torriani et al., 2009). Our results showed that G143A has increased over five years, reaching over 70% in Lithuania and 50% in Estonia (not detected in Estonia in 2014) and Latvia. Only in Finland, the frequency of this mutation decreased slightly during this period (Table 3). G143A mutants are known to have a high level of cross-resistance between different strobilurins. Even though not recommended anymore against STB, QoI fungicides remain effective against other diseases (rust diseases) and are therefore still applied in the field. The continuous use of any strobilurin creates favorable conditions for the further spreading of *Z. tritici* strains carrying G143A.

Field isolates with reduced sensitivity to SDHI fungicide commonly carry a single amino acid substitution in one of the four *Sdh* subunits (Scalliet et al., 2012). In this study, the average EC_50_ values varied in the region from 0.07 to 0.33 ppm. Only a few strains surpassed the EC_50_ of 1.0 ppm. In Ireland, reduced field efficacy of SDHI was especially correlated with a high frequency of C-T79N (Kildea, personal communication). In Scandinavia, only rare cases of C-T79N and C-N86S are reported each year. No *Sdh* mutations were detected in the isolate collection tested in this study. *Sdh* mutations are, thus, not drivers for increased tolerance in these isolates. These findings are similar to the results presented by Yamashita and Fraaije (2018). The appearance of resistant isolates in the populations can be the result of *in planta* degradation of SDHI caused either by the plant and/or the fungus itself. This study shows that adapted *Z. tritici* strains exist in the fields and should be considered a potential risk for the future.

Finally, we did not detect any MDR strain among our collected isolates. Interestingly, using the PCR diagnostic test to detect MDR strains as proposed by Omrane et al. (2015), we found one strain that harbors a previously unknown insertion in the *MFS1* promoter. Since this strain does not display an MDR phenotype, we suspect that it does not overexpress the *MFS1* gene. It is intriguing to notice that this is the fourth type of repeated insert detected in the *MFS1* promoter. While the three other insertions are or may be putative relics of TE driving *MFS1* overexpression, the insertion of a whole transposon observed in strain 18-Zt-EE-06-03 probably abolishes transcription of *MFS1*, especially since the transcription of the transposase is from the opposite strand. The frequent presence of repeated elements in the *MFS1*, but also in the *CYP51* promoter (Chassot et al., 2008; Cools et al., 2012; Kildea et al., 2019) raises the question of promoter plasticity. Additional population genomic studies are required to clarify if these types of the insert are distributed uniformly over the entire *Z. tritici* genome, or if some genomic regions, such as specific promoters, are preferred. Moreover, the role of fungicide selection pressure in promoter insertions remains to be evaluated.

## Conclusion

In conclusion, the results presented in this study show an increase in EC_50_ values of the Baltic-Finnish *Z. tritici* population of commonly used azoles. We showed that the frequencies of key *CYP51* mutations and G143A of the Baltic-Finnish *Z. tritici* population have increased from 2014 to 2018. The results indicate that the same development has taken place in the region, as had been witnessed in other European regions. Resistant management strategies should be advocated to prolong the field efficacy of all MoA used against STB. Though no SDHI resistance-conferring mutations were detected, the possible existence of non-target site SDHI resistance should be considered for the design of resistance management strategies. Finally, we found a strain harboring a fourth transposon insertion in the promoter of the *MFS1* gene, which, however, does not display an MDR phenotype.

## Data Availability Statement

The datasets generated for this study can be found in the https://www.ncbi.nlm.nih.gov/nuccore/mn813065.

## Author Contributions

AM and TH performed the experiments and analyzed the data. SF analyzed the data on the *MFS1* insert. AM, SF, TH, and PS drafted the manuscript. All authors read and approved the final manuscript.

## Conflict of Interest

The authors declare that the research was conducted in the absence of any commercial or financial relationships that could be construed as a potential conflict of interest.

## Footnotes

1 https://blast.ncbi.nlm.nih.gov

2 emboss.sourceforge.net/
